# Cellnet technology to generate 3D, functional, single-cell networks in custom architectures within collagen

**DOI:** 10.1088/1758-5090/adc48f

**Published:** 2025-04-03

**Authors:** Arun Poudel, Puskal Kunwar, Ujjwal Aryal, Anna-Blessing Merife, Pranav Soman

**Affiliations:** Department of Chemical and Biomedical Engineering and BioInspired Institute, Syracuse University, Syracuse, NY 13210, United States of America

**Keywords:** CELLNET, 3D, technology, spatially, organized, functional

## Abstract

Cells possess the remarkable ability to generate tissue-specific 3D interconnected networks and respond to a wide range of stimuli. Understanding the link between the spatial arrangement of individual cells and their networks’ emergent properties is necessary for the discovery of both fundamental biology as well as applied therapeutics. However, current methods spanning from lithography to 3D photo-patterning to acoustofluidic devices are unable to generate interconnected and organized single cell 3D networks within native extracellular matrix (ECM). To address this challenge, we report a novel technology coined as Cellnet. This involves the use of natural collagen crosslinked within three-chambered microfluidic chips followed by femtosecond laser-assisted cavitation to generate user-defined 3D microchannel networks. Model cells, seeded within side chamber of the chip, migrate within microchannel networks within hours, self-organize and form viable, interconnected, 3D single-cell networks in custom architectures such as square grid, concentric circle, parallel lines, and spiral patterns. Heterotypic Cellnets can also be generated by seeding multiple cell types in side-chambers of the chip. The functionality of cell networks can be studied by monitoring the real-time calcium signaling response of individual cells and signal propagation within Cellnets when subjected to flow stimulus alone or a sequential combination of flow and biochemical stimuli. Furthermore, user-defined disrupted Cellnets can be generated by lethally injuring target cells within the 3D network and analyzing the changes in their signaling dynamics. As compared to the current self-assembly based methods that exhibit high variability and poor reproducibility, Cellnets can generate organized 3D single-cell networks and their real-time signaling responses to a range of stimuli can be accurately captured using simple cell seeding and easy-to-handle microfluidic chips. Cellnet technology, agnostic of cell types, ECM formulations, 3D cell-connectivity designs, or location and timing of network disruptions, could pave the way to address a range of fundamental and applied bioscience applications.

## 1. Introduction

**Figure 1.**
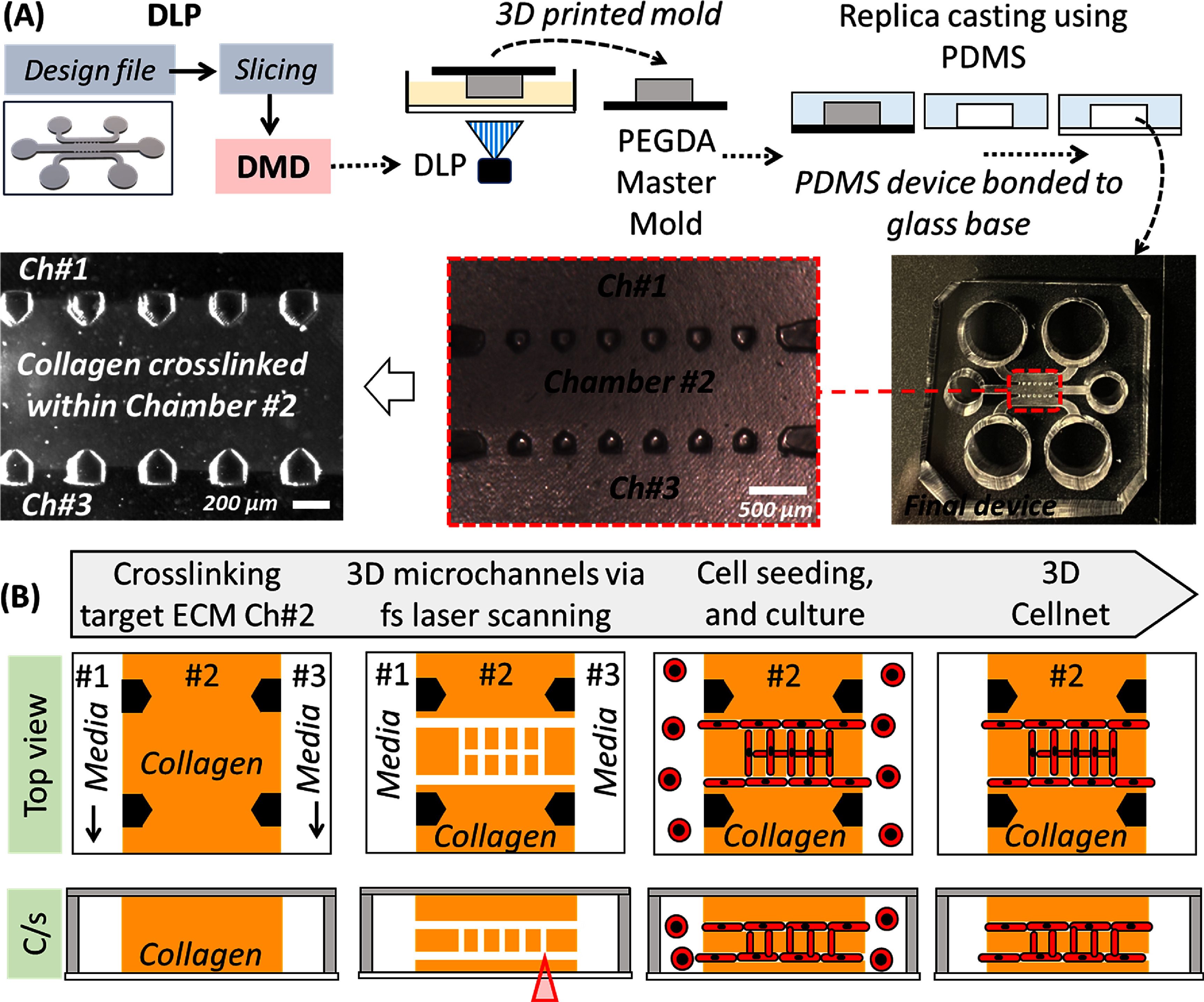
(A) Schematic showing 3D printing of master molds using digital micromirror device (DMD) based digital light projection (DLP) followed by replica casting to generate three-chambered microfluidic chips using PDMS (picture). Type I collagen solution is perfused and crosslinked within chamber #2. (B) Process flow with top and cross-sectional views to generate a two-layer interconnected cellular networks or Cellnets within Type I Collagen in the central chamber of the microfluidic chip.

Recreating the 3D spatial organization of single-cell networks will help elucidate underlying mechanisms related to their emergent functional properties exhibited at the tissue level. Although technological advances have led to an array of methods to arrange cells in 2D and 3D to mimic tissue specific architectures, single-cell resolution with control over cell-to-cell connectivity remains challenging [1, 2]. A common strategy is to pattern 2D adhesive micropatterns using conventional lithography methods followed by cell seeding to generate interconnected single cell networks. Other methods such as laser-assisted bioprinting has also been used to directly deposit single cells and generate 2D cellular arrays, however, arranging single cells in 3D has proved difficult [3, 4]. Methods based on 3D photo-patterning of adhesive peptides within specialized semi-synthetic hydrogels have emerged [5], but they have failed to realize 3D single cell networks. At present, only methods based on multi-photon absorption can pattern features at single-cell resolutions [6–15] although limitations related to specialized photosensitive materials, water soluble low-toxicity photoinitiators, cell viability during laser scanning in the presence of cells, and low scalability, have limited its use in the field. Thus, the field continues to rely on self-assembly based methods, that involve mixing relevant cells in natural extracellular matrix (ECM) like collagen or fibrin. This however results in randomly organized 3D cell networks without precise control over network density, connectivity, and architecture, making systematic mechanistic study on networks’ properties challenging. Bioprinting methods can provide spatial control over cell placements within 3D ECMs [16–20], however single cell resolution is not possible [21]. Microfluidic devices integrated with acoustic [22–24], dielectric [25, 26], and magnetic field stimulation [26, 27] have also been used to directly manipulate cells in a contactless manner. However, these methods cannot achieve single-cell resolution or user-defined multi-layer 3D patterns, and its control over intercellular connectivity remains poor. In summary, current methods are unable to generate tissue-specific, 3D, single-cell networks within natural, unmodified ECM, like collagen. To address this challenge, we report a new technology coined as Cellnet to generate 3D, single-cell, functional networks in custom architectures within multi-chambered chips using microchannel patterns within collagen generated using laser-assisted cavitation (figure 1). First, digital light projection (DLP) was used to rapidly design and print master molds which were used to generate custom three-chambered PDMS devices. Second, ECM of interest (type I collagen) was perfused into central chambers and thermally crosslinked to generate a barrier between chambers 1 and 3. Third, femtosecond laser assisted cavitation was used to generate 3D microchannel patterns within collagen. Model cells, seeded within the device, self-assemble within microchannel patterns, and generate an interconnected, 3D, functional circuits coined as Cellnets. We show that Cellnets are compatible with standard imaging methods (brightfield, immunostaining, time-lapse microscopy), co-culturing cells and *in situ* manipulation such as application of fluid flow and/or biochemical stimuli, or injury to target cells to generate user-defined disrupted networks. Cellnet, generated using tissue-specific cells, matrix, and spatial organization, has the potential to emerge as a deterministic model to study how short-term signaling results in durable functional or yet unknown emergent properties.

## 2. Results

### 2.1. Design and development of microfluidic chips using DLP-printed Master Molds

We used DLP to print master molds which were used to replica cast final PDMS microfluidic chips (figure 1(A) and SI-figure 1(A)). Details related to the DLP printing setup, pre-polymer formulation, printing conditions, glass modification protocols, are reported in the SI section. Briefly, a CAD model of a reverse master mold of the intended microfluidic chip was designed in SolidWorks; this consist of positive features that would be replicated into three microfluidic chambers with negative spaces, each with inlet and outlet ports (SI-figure 1(B)). CAD model was then sliced to generate a series of virtual masks which were converted to corresponding light patterns by the digital micromirror device. Upon irradiation onto liquid PEGDA (250 MW) photo-polymer solution in a layer-by-layer manner, final master mold was realized. Glass surface was modified to prevent delamination of PEGDA mold during the printing process. Molds were printed at the step size of 20 *µ*m with 0.8 s exposure time per layer with constant light intensity of 6 mW cm^−2^. Post-printing, molds were cleaned with 100% ethanol to remove any uncured resin. Molds were cured for an additional 20 s under UV light to ensure strong crosslinking between the mold and the bottom glass slide to prevent any delamination during the PDMS casting steps. PDMS prepolymer solution was casted onto the molds followed by thermally curing at low and high temperature cycles; this ensured high repeatability, since without the low temperature curing step, molds show tendency to crack and warp. Prior to casting, PEGDA molds were submersed in 100% ethanol for an hour followed by exposure to ambient light for 24 h to generate defect-free PDMS devices. Without these post-processing steps, PDMS casting led to many defects possibly due to leaching of free radicals from the interior of the mold causing inhibition of PDMS crosslinking at the mold-liquid PDMS interface [28] (SI-figure 1(C)). Polymerized PDMS was carefully peeled off, trimmed to suitable size, and 6 inlet-outlet holes were punched (2 holes per chamber) before irreversibly bonding to a glass coverslip (0.15 mm thick) to generate final PDMS devices (figure 1(A)). The inlet and outlet holes for the central chamber were punched using 3 mm biopsy punches, while those for the side chambers were punched using 5 mm biopsy punches. The total height of the chip was 4.5 mm while the height for all three chambers in the chip was 240 *µ*m. The final devices consist of three-chambers: a central chamber (Ch#2; 1 mm wide, designed to house crosslinked collagen) flanked on either side by two chambers (Ch#1 and Ch#3; 1 mm wide, for media exchanges). These chambers are separated by an array of microposts with a width of 200 *μ*m and a spacing of either 150 *μ*m or 200 *μ*m. Chips with micropost spacing of 150 *μ*m were used to design custom Cellnet, while chips with micropost spacing of 200 *μ*m were used for calcium signaling experiments as the larger spacing accommodates a higher number of cell networks.

### 2.2. Characterization of 3D microchannel networks within crosslinked collagen generated using laser-assisted cavitation

**Figure 2.**
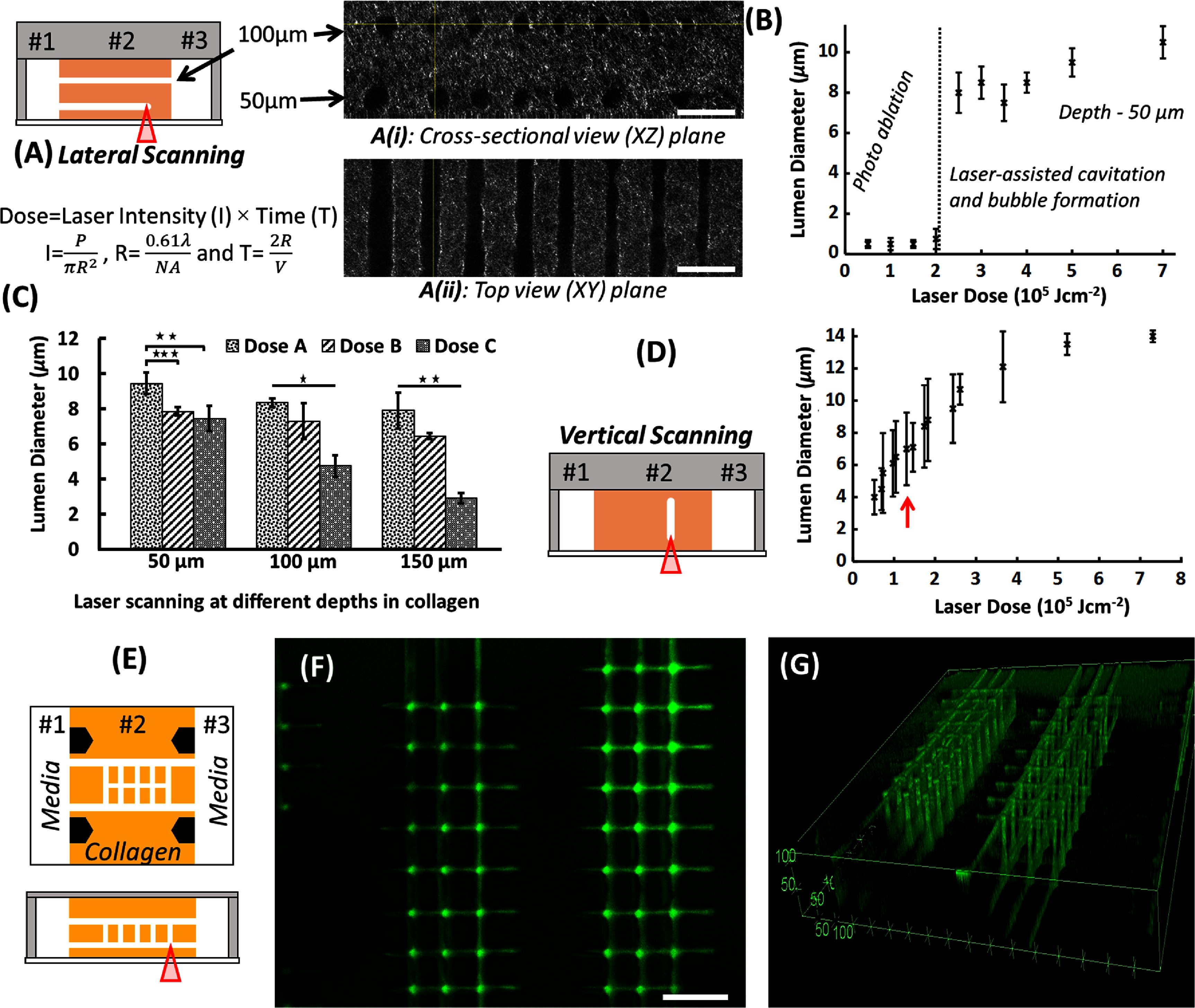
Characterization of microchannels generated within collagen. (A) Schematic showing lateral scanning (*XY*) with varying dosage, and reflectance confocal images of microchannels. Scale bar: 25 *μ*m (B) During lateral laser scanning, plots show the relationships between lumen diameter and laser dosage at a depth of 50 *µ*m, while (C) shows the effect of varying depth and laser dosages (Dose A: 7.5 × 10^5^ Jcm^−2^, Dose B: 4 × 10^5^ Jcm^−2^, Dose C: 2.5 × 10^5^ Jcm^−2^) on lumen diameter. (Error bars: Mean ± SD; *p < 0.05; **p < 0.01, and ***p < 0.001) (D) Schematic and plot showing vertical scanning to generate microchannels and the relationship between lumen diameter and laser dosage. (E) Schematic of a 3D microchannel grid with lumen diameter of ∼8 *µ*m in *XY* (lateral) and *Z* (vertical) directions within crosslinked collagen in chamber #2 of a three-chambered microfluidic chip. (F)–(G) Top and isometric view of confocal images after perfusion of a solution of fluorescent microbead (*φ* = 0.5 *µ*m) into the microchannel network. Scale bar: 100 *μ*m.

**Figure 3.**
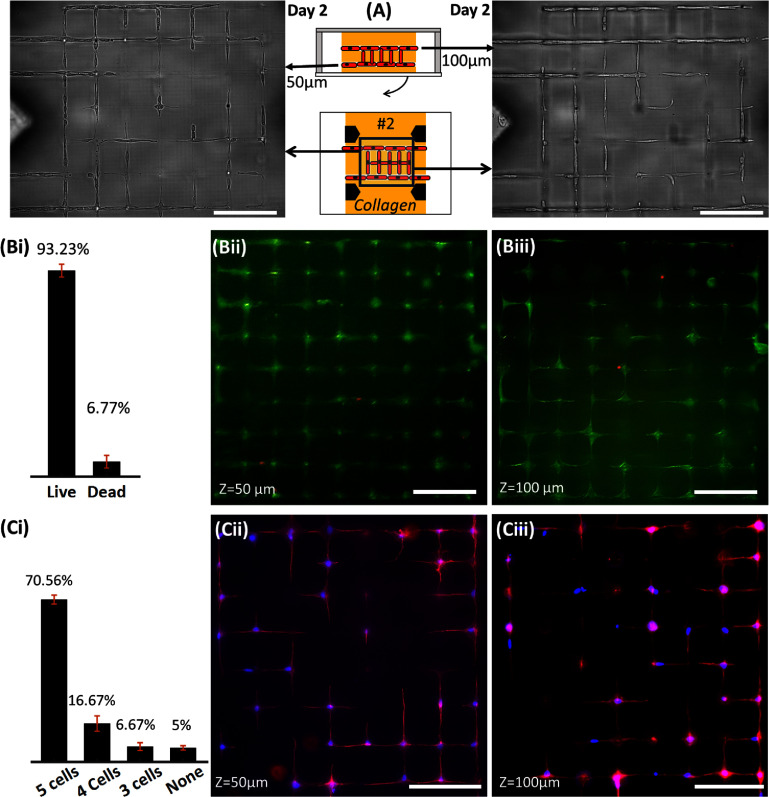
(A) Schematic and representative brightfield images from two *z*-planes (located at 50 *µ*m and 100 *µ*m from the bottom glass surface inside collagen matrix) showing seeding of model cells (10T1/2s) in chamber #1 or #3, and their migration within ablated networks to form viable, interconnected, 3D single-cell networks or Cellnets. Viability plot (B-i) and live/dead assay (B-ii), (B-iii) of Cellnets on Day 7 taken from both *z*-planes of the 3D network show live cells (Green, calcein) and dead cells (ethidium homodimer-1). (C) Connectivity plot ((C-i), Day 7) and cell morphology at two different *z* planes (C-ii), (C-iii) (actin = red; nucleus = blue). Scale bar: 100 *μ*m.

In this work, we choose type I collagen as model ECM due to its abundance in *in vivo* tissues, and wide use in the field to generate 3D cell culture models. Before pipetting collagen solution within PDMS chips, chips were surface modified to prevent delamination of crosslinked collagen from the channel surfaces during active cultures. First (3-aminopropyl)triethoxy silane was used to silanize the glass/PDMS surfaces to generate self-assembled monolayer (SAM) with reactive functional groups (–NH_2_) followed by glutaraldehyde to form reactive functional groups (–COOH) [29]. Without these modification steps, the crosslinked collagen showed a tendency to detach from the PDMS roof and glass bottom surfaces (SI-figure 1(D)). Then, type I collagen solution (4 mg ml^−1^) was crosslinked within the central chambers of UV sterilized chips (figure 1(B)). After 24 h, a focused femtosecond laser (800 nm, 1.2 W; the laser setup is shown in SI-figure 2(A)) was used to generate 3D microchannels architectures. To reliably generate 3D channels of defined lumen sizes, laser scanning was performed at varying power and depths inside collagen (50–200 *µ*m) in both lateral (*XY*) (figure 2(A), SI-figure 2(B) and video 1) and vertical (*Z*) (figure 2(D), SI-figure 2(C) and video 2) directions. Reflectance microscopy images show top and cross-sectional views of microchannels embedded in collagen matrix. Laser dosage below 2 × 10^5^ Jcm^−2^ results in microchannels with lumen size of 0.5–1 *µ*m, while above this threshold, laser irradiation results in cavitation and formation of larger sized lumens (8–10 *µ*m) (figure 2(B)). During laser scanning above this threshold, the radial expansion of the bubble generates a shockwave which locally breaks down the collagen network and leaves behind a hollow lumen in its wake (SI-figures 3(A), (B) and video 7). We also noticed that gas bubbles were elongated in the scanning direction and often remain in the channel from 10 s of seconds to 1–2 h. We found that if laser was irradiated on collagen immediately after thermal crosslinking, then scanning induced bubble collapses and the channels close, possibly due to weak mechanical properties of partially crosslinked collagen. Thus, 24 h were given for completion of collagen crosslinking before laser irradiation was performed; this ensured repeatable and stable channel formation without collapse (SI-figure 3(D)). For constant laser dosage during lateral scanning, lumen diameter decreased with increased depth in collagen (figure 2(C)). Laser processing plots (shown in figures 2(B) and (D)) were used as a design guide to repeatably generate 3D microchannel networks with a *XY* lumen diameter of ∼8 *µ*m and *Z* lumen diameter of ∼6–8 *µ*m within collagen; this lumen diameter was found to be ideal for single cell migration in the horizontal lumen networks. Lumen diameter >8 *µ*m led to migration of multiple cells within the channels while lumen diameter <8 *µ*m decreased single cell migration in the microchannel network. For this work, we used 4 mg ml^−1^ collagen concentration with a laser power of 1.4 W and 40× water objective (0.8 NA) to generate various architectures of Cellnets. Fluorescent beads (∼0.5 *μ*m) were used to verify perfusion within 3D microchannel network within the central chamber (Ch#2) of a microfluidic chip (figures 2(E)–(G)).

### 2.3. Generation of viable and interconnected single-cell 3D networks within microfluidic chips

**Figure 4.**
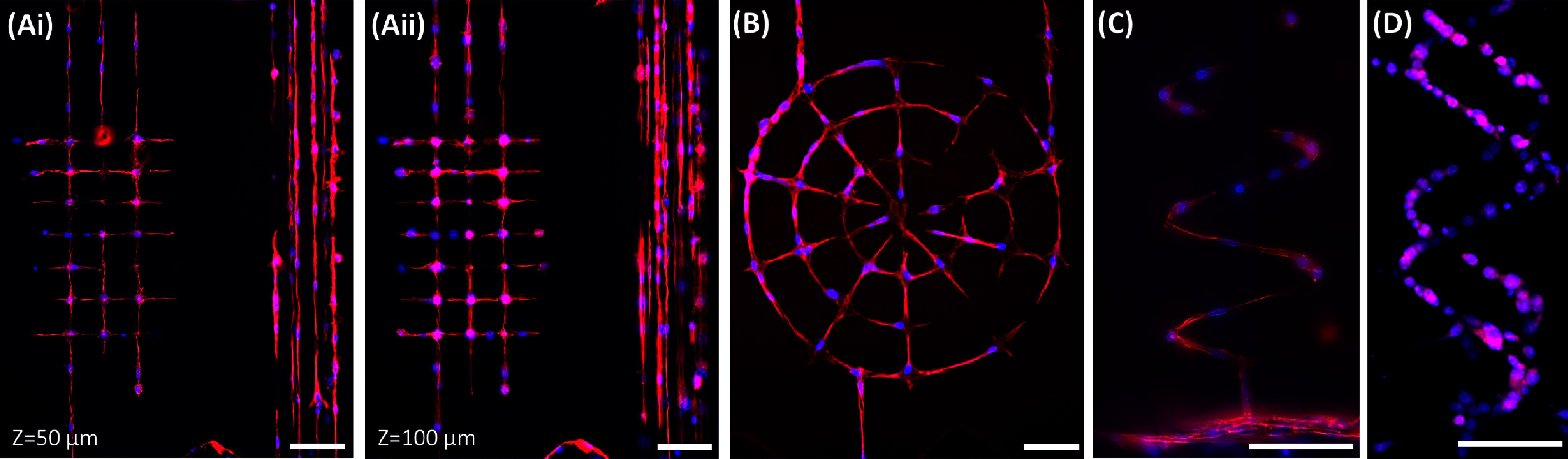
(A) Representative images showing single cells organized within user-defined square grid and parallel line architectures in two different *z*-planes within collagen matrix. Representative image of cell morphology organized in various patterns (B) concentric rings, (C) out-of-plane spiral and (D) double-helix. Scale bar: 100 *µ*m. (Actin: Red; Nucleus: Blue).

We used fibroblast-like model 10T1/2 cells to demonstrate the formation of Cellnets. First, cell solution (1 M ml^−1^) was pipetted in one of the side chambers (Ch#1 or Ch#3) of the microfluidic chip, and brightfield microscopy was used to monitor cell migration within the microchannel network. We observed migration as early as 3 h post-seeding. Between Day 1-2, cells migrated inside the collagen (500–1000 *μ*m from the interface between Ch#2 and Ch#1/3), and assemble into a 3D, interconnected single cell network within chamber #2 of the microfluidic chip (figure 3(A) and SI-figure 4(A)). On Day 2, extra cells were flushed out using 0.25% trypsin treatment for 5 min followed by pipetting fresh media in the side chambers; this step was performed to prevent aggregation of cells in Ch#1 or Ch#3 that could block diffusion of reporter dyes or antibodies and generate unwanted fluorescence during the characterization of cell networks. During this step, loss of cells, especially from areas of lumen in collagen that are open to side chambers, cannot be ruled out. We observe when Cellnets were subjected to the trypsin washing step, individual cells within the network tend to detach from the lumen walls and dendritic extensions were retracted for a few hours before recovering back to their normal spread morphology after 12 h (SI-figure 4(B)). Single cells continue to occupy the lumen microchannel network even after this washing step, although migration of cells within the network or out of the network in the side chambers were not characterized. We note that this trypsin washing step is necessary for noise free and repeatable imaging of single-cell network. To evaluate viability and morphology of cells within Cellnets, a 3D microchannel network with a two-layer connected grid architecture was used. The upper- and lower-layers, located 100 *µ*m and 50 *µ*m respectively from the glass bottom substrate, connect with each other via vertical lumens. For this work, the lumen diameter for vertical scanning was chosen to be ∼6–8 *µ*m (red arrow, figure 2(D)). Interestingly, we observed a preference of cell nuclei to reside within the *XY* (lateral) lumen at the nodes (intersection of two laser scanning paths) as compared to the vertical lumens, possibly because of their small size (diameter ∼6–8 *µ*m) as compared to lateral lumens (diameter ∼8 *µ*m), although the exact reason cannot be ascertained from this work.

**Figure 5.**
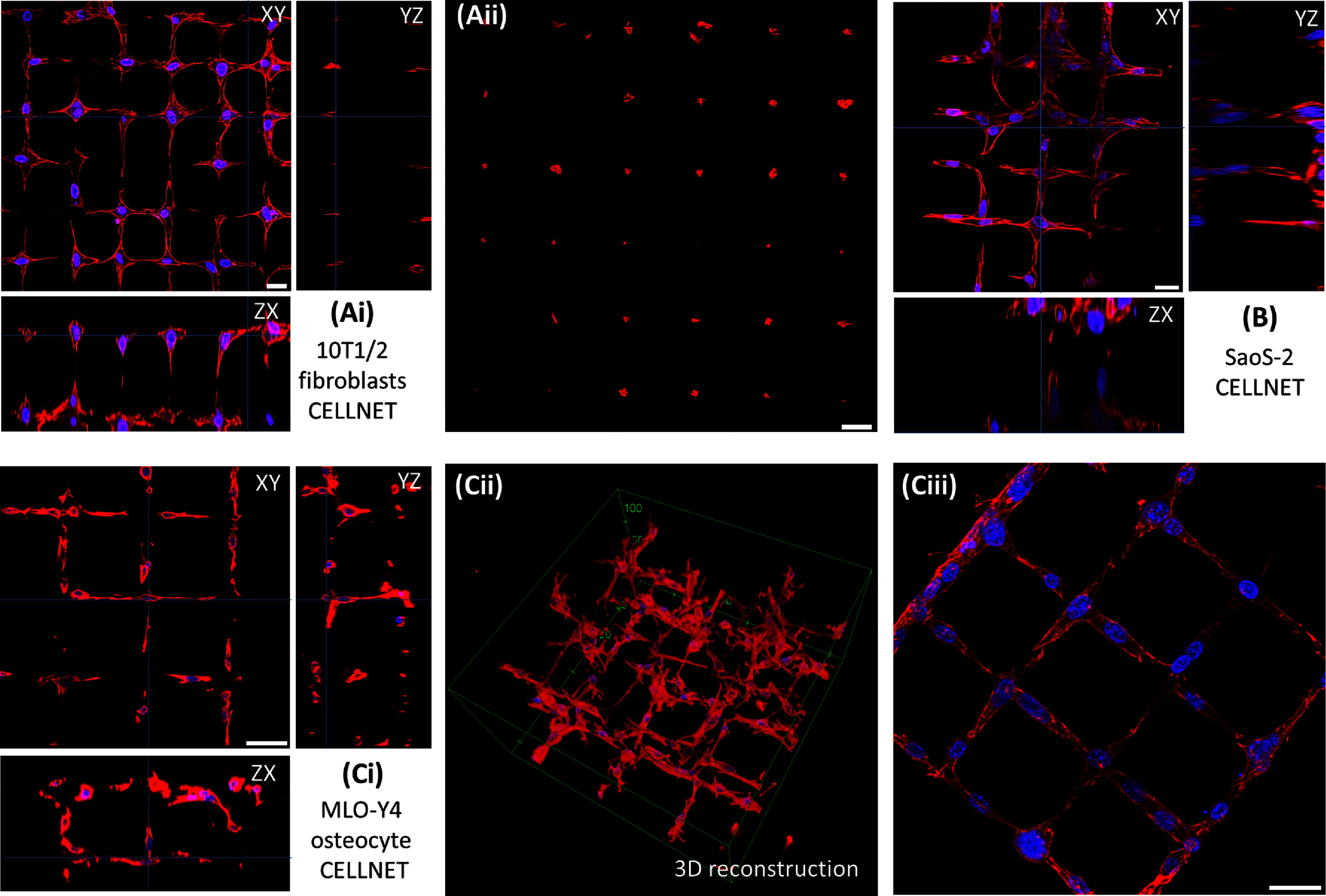
(A)–(C) Morphology of Cellnets using three model cells. (A-i) Orthogonal views showing 10T1/2 Cellnet’s 3D morphology. (A-ii) Cross-sectional image showing actin-labelled cellular processes extending between two *z*-planes ∼50 *μ*m apart. (B) Orthogonal views showing 3D SaoS-2 Cellnet. (C-i) Orthogonal views showing 3D MLO-Y4 Cellnets, (C-ii) 3D reconstructed image and (C-iii) high-resolution image of MLO-Y4 Cellnets on one plane. Scale bar: 25 *μ*m. (Actin: Red; Nucleus: Blue).

**Figure 6.**
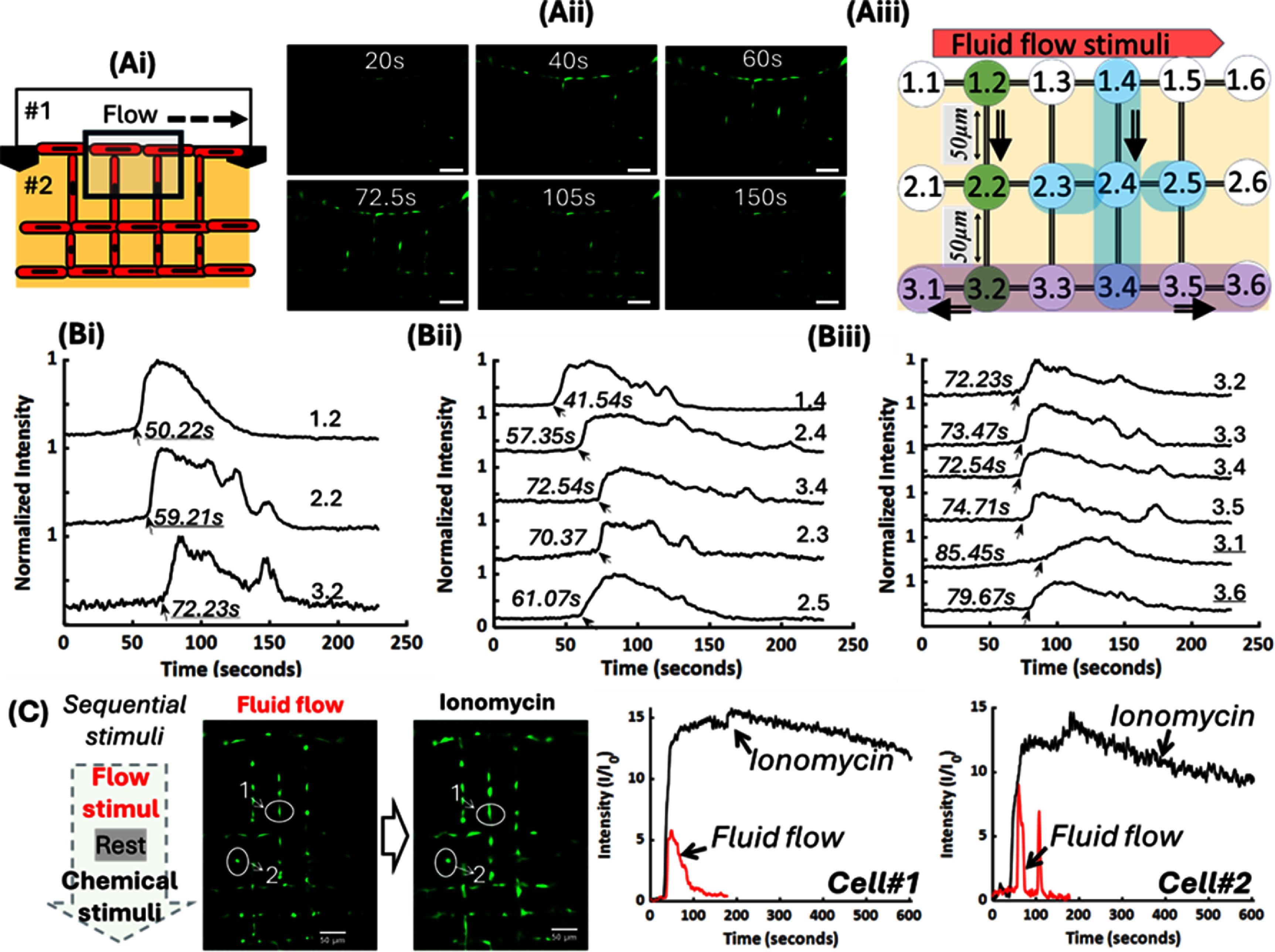
Real-time signaling within osteocyte (MLO-Y4) Cellnets. (A-i) Schematic showing application of a biophysical fluid flow stimuli in Chamber #1. (A-ii) Time-lapse fluorescence images showing calcium signaling at specific time-points. (A-iii) Schematic of model Cellnet. Here, 1.2 represents a single cell in row 1 and column 2, located at the interface of Ch#1-2. Upon stimulation, 1.2 initiates a Ca signal that propagates to adjacent connected cells embedded deeper within collagen (2.2 and 3.2) (B-i)–(B-iii) Normalized single cell signals from three representative local networks (time-stamps are shown in seconds). (C) Signaling response of individual osteocytes (Cell #1 and #2) and the entire network can be accurately analyzed in the presence of sequential biophysical and biochemical stimuli. Scale bar: (A-ii), C: 50 *µ*m.

Viability of cells, calculated from both layers, was found to be 93.23 ± 2.87% (figures 3(B-i) and (B-ii, B-iii)). Fluorescence image of cells fixed on Day 7 shows morphology (actin/nucleus) of cells assembled into pre-templated square-grid microchannel networks that provide defined control over cell-to-cell connectivity within collagen (figures 3(C-i) and (C-ii, C-iii)). For instance, in the template used here, for a target cell within the Cellnet, a maximum of 5 cell-cell connections can be generated: 4 in-plane connections (in either the upper or lower layers) and one-out of plane (between the two layers). Results show that 70.56 ± 1.92% cells were connected to 5 cells, while 16.67 ± 3.33% were connected to 4 cells, 6.67 ± 1.67% were connected to 3 cells, while 5 ± 0.96% were not connected to any cells (figure 3(C-i)). This shows that around 95% cells within Cellnets are connected to at least three neighboring cells. SI-figure 5 shows composite brightfield and fluorescence images, and a reconstructed 3D image. Results show variability in the number of single-cell connections based on cell types. For instance, 10T1/2-Cellnet exhibit 5, 4, 3 and 0 cell-cell connections (figure 3(C-i)) while MLO-Y4s show all possible cell-to-cell connections (0–5) (SI-figure 6(B-ii)). To demonstrate that Cellnets of complex topology can be generated, we designed microchannel templates such as parallel lines, spider webs, out-of-plane spirals, and double helixes (figure 4). Results show that cells migrate and self-organized into single cell 3D networks in custom orientations.

**Figure 7.**
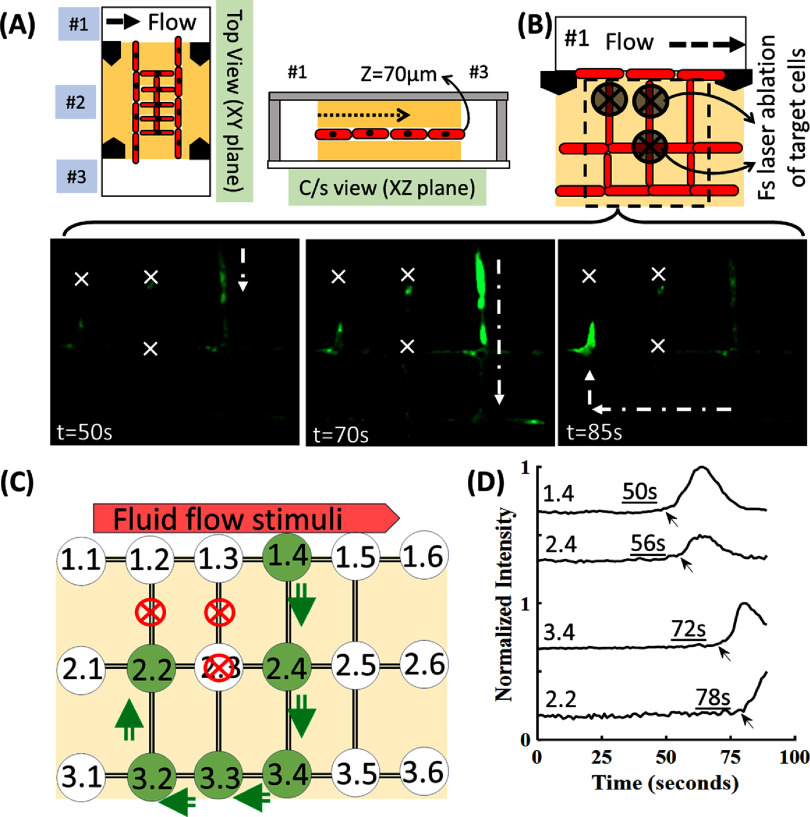
In-plane (∼70 *µ*m inside collagen) real-time signaling within user-disrupted osteocyte Cellnets. (A) and (B) (i) schematic showing application of a biophysical fluid flow stimuli in chamber #1 after femtosecond laser irradiation was used to lethally injury three cells (shown by cross mark). (C) Simplified representation of how signal propagation is diverted due to targeted disruptions. (D) Intensity plots showing delayed propagation of signal to cell 2.2 as signal travels from cell 1.4 to 2.4 to 3.4 to 2.2. Scale bar: (B): 50 *µ*m.

To demonstrate that Cellnets can work with many cell types, we generated 3D square-grid microchannel templates within collagen, and seeded fibroblasts-like 10T1/2s, MLO-Y4 osteocytes, and Saos-2 osteoblast-like cells. Top, side and 3D reconstructed views of Cellnets show formation of Cellnets with in-plane and out-of-plane connections (figures 5(A)–(C)). Figure 5(A-ii) highlights a cross-sectional section ∼50 *µ*m from the bottom glass substrate—a plane between the upper and lower microchannel grids showing ∼90% of out-of-plane actin-labelled cellular connections that span ∼50 *µ*m distance in the vertical direction. More cells tend to occupy the intersecting lumen regions possibly due to its slightly larger volumes. We found that cell connectivity is a function of lumen size and cell type, and this has to be optimized to get a single cell connectivity close to 100% (SI-figures 6(A-iii) and (B-ii)). For instance, for MLO-Y4 Cellnets, an ablated channel size of ∼8 *µ*m ensuring single cell occupancy with ∼85% cell-to-cell connectivity while a lumen size of ∼12 *µ*m increases connectivity to ∼97% at the cost of having more than one cells in the larger microchannels. Furthermore, we tested the capability of generating Cellnets using two different cell types by seeding cells tagged with different fluorescent dyes on either side of the central chamber (Ch#2) (SI-figure 7). A 3D square-grid microchannel network was generated within collagen. One study involved fluorescently tagging of the same cell type (MLO-Y4) with green and red dye, while the other study tagged MLO-Y4 with green dye and pre-osteoblasts (MC3T3) with red dye. In both cases, cells migrate towards each other within the 3D microchannel network and form heterotypic Cellnets.

### 2.4. Testing functionality of Cellnets using stimuli evoked real-time Calcium signaling studies

**Figure 8.**
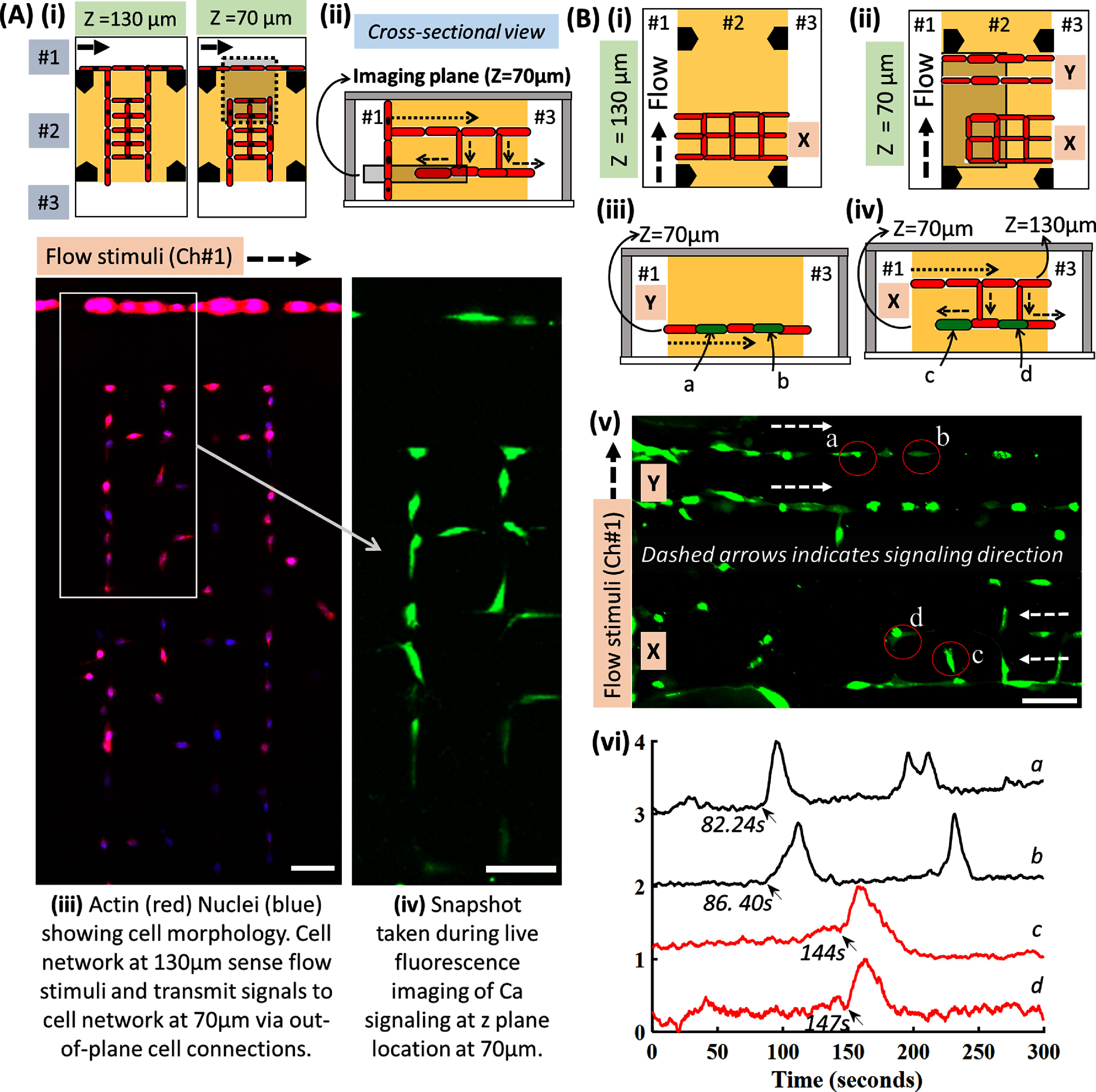
Out-of-plane (3D) real-time signaling within user-disrupted osteocyte Cellnets. (A) Schematic of 2-layered network with cells organized using a grid patterns (*X*). Top views (i) and cross-sectional view (ii) showing out-of-plane signal propagation. (iii) Actin (red) Nuclei (blue) showing cell morphology. Cell network at 130 *µ*m sense flow stimuli and transmit signals to cell network at 70 *µ*m via out-of-plane cell connections. (iv) Snapshot taken during live fluorescence imaging of Ca signaling at *z* plane location at 70 *µ*m. (B) Schematic of 2-layered network with two different cell patterns (*X* = grid, *Y* = parallel line) located near each other at (i) *z* = 130 *µ*m and (ii) at 70 *µ*m in the same sample. Cross-sectional views for (iii) *Y*-pattern and (iv) *X*-pattern showing out-of-plane signal propagation at different planes.(v) Snapshot taken at a *z*-plane of ∼70 *µ*m during calcium signaling experiment. Cells *a, b* in pattern *Y* and *c, d* in pattern *X* have been marked by a red circle, while dashed white arrows indicate signal propagation directions. (vi) Signal intensity peaks of cells ‘*c*’ and ‘*d*’ in pattern *X* are delayed as the signal travels from the cell-network @*z* = 130 *µ*m to cell-network @*z* = 70 *µ*m as compared to cells ‘*a*’ and ‘*b*’ in pattern *Y* where flow stimuli have a direct path via cell network @*z* = 70 *µ*m. Note: In pattern *Y*, cells are directly exposed to flow stimuli while in pattern *X*, only the top layer is directly exposed to stimuli.

Since larger lumen size (∼12 *µ*m) tend to house multiple cells in the same location, (SI-figure 6(A-iv)), we chose a lumen size of ∼8 *µ*m and a square grid network design to test signaling within MLO-Y4 Cellnets that mimics an organized 3D osteocyte interconnected networks found within bone tissue. Here we studied the real-time signaling responses of individual cells and the entire network when Cellnets are subjected to biophysical and biochemical stimuli. Before monitoring of real-time signaling within Cellnets, the viability and morphology of osteocytes were analyzed. Results show an organized square grid network of cells with high viability of 91.39 ± 1.08%. (SI-figure 6(A)) Osteocytes form 3D in-plane and out-of-plane cell-to-cell connections as evidence by the 3D reconstruction and orthogonal views. As explained earlier, number of cell-to-cell connections were characterized (SI-figure 6(B-ii)). Specifically, 22.45 ± 2.04% cells connect with 5 neighboring cells (4 in-plane and 1 out-of-plane connections), 20.07 ± 0.59% connect with 4 cells, 25.17 ± 2.36% connect with 3 cells, 11.56 ± 1.56% with 2 cells, 5.44 ± 3.28% with one cell, while 15.31 ± 1.77% were not connected to any neighboring cells. Thus, for this 2-layer design, we found that ∼85% of cells were connected to at least one neighboring cell within the network while maintaining single cell occupancy within ablated channels. First, MLO-Y4 Cellnets were generated in the central chambers (Ch#2) of microfluidic chips and treated with Fluo-4 Ca indicator via media chambers (Ch#1, Ch#3) (figure 6(A-i)). Timelapse fluorescence images show an increase in calcium signals in cells proximal to the flow stimuli, and subsequent transmission of the signals to cells embedded deeper within collagen (figure 6(A-ii)). To track signaling response of individual osteocytes within Cellnets, a simplified nomenclature ‘*x.y*’ was used, where *x* points to row number and *y* points to column number (figure 6(A-iii)). For instance, row 1 represents osteocytes at the interface of central and side chamber that could directly experience the stimuli, while rows 2 and 3 represent osteocytes embedded at increasing distance within collagen (away from the stimuli). Control over laser scanning path allows the generation of a network with deterministic cell-to-cell connectivity. For instance, in figure 6(A-iii), a black line represents a direct microchannel connection between cell 1.1 and embedded cell 1.2 while an absence of a black line between cell 1.1 and cell 2.1 indicates that these cells are not connected. For individual cells within the network, calcium signaling, expressed as fold change in fluorescence over baseline, was plotted and time-lags between locally connected osteocyte circuits were analyzed (figures 6(B-i) (B-ii) and (B-iii)). For instance, signals travel from cell 1.2 to 2.2 to 3.2 (a distance of ∼100 *µ*m) with peaks recorded at 50.22 s, 59.21 s and 72.23 s respectively (total time of 22.01 s for a ∼100 *µ*m resulting in speed of ∼4.54 *µ*m s^−1^) (figure 6(B-i)). This process was repeated for multiple local cell circuits within Cellnets to obtain an average signal propagation velocity of 4.55 ± 0.26 *μ*m s^−1^ (SI-figure 8). Each cell within a 3D connected network function as a node, which when connected to other cells in the network, relay stimuli evoked Ca signals. Consider another locally connected network (marked in blue), here a clear time delay is seen as signal travels from cell 1.4 (41.54 s) to 2.4 (57.35 s) to three connected cells 3.4 (72.54 s), 2.3 (70.37 s), and 2.5 (61.07 s) (figure 6(B-ii)). Since all cells are not always present at the nodes with equal spacing between them, the location of each cell was analyzed using FIJI image processor to get accurate measurements about propagation speeds (SI-figure 8). Lastly, consider all cells in third row; here cells 3.1 and 3.6 are not connected to cells from previous rows, while cells 3.2, 3.3, 3.4 and 3.5 are connected to cells in row 2. As expected, the signal travels to the connected cells first before propagating to 3.1 and 3.6 via 3.2 and 3.5 cell-cell connections respectively (figure 6(B-iii)).

We then provide a sequential flow stimulus, followed by rest, followed by a chemical stimulant (ionomycin, 5 *μ*l, 45 *μ*mol·l^−1^, commonly used ionophore known to enhance Ca signals) (figure 6(C)). The sequence of fluid flow, rest, and ionomycin stimulus was performed on the same sample, and resulting changes in calcium flux were captured. Here we pick two osteocytes (marked as #1, #2) within the Cellnet and monitored their response over time when the network is subjected to a defined set of sequential stimuli. Results show signaling heterogeneity in magnitude and signal profiles between the two cells and between the stimuli type for the same cell. SI-figure 9 shows calcium intensity plots for 15 individual osteocytes within the network. Like earlier results, cells closest to the stimuli are triggered first, followed by signal propagation inside the entire network. Time delays within the network is directly related to the user-templated microchannel design and associated cell-to-cell connectivity.

### 2.5. Study of calcium signaling and signal propagation within custom designed disrupted Cellnets

Here, we show the Cellnets can be disrupted in custom architecture by ablating individual cells within the network by targeted femtosecond laser irradiation. First, laser threshold to lethally injure target osteocytes was identified using standard live/dead assay. Osteocyte monolayer, labeled with green cell tracker, were identified, and irradiated with femtosecond laser at varying power (10–200 mW) and exposure times (0.5–1 s). Laser threshold to cause permanent injury was identified to be 0.22 J cm^−2^. To compensate for a decrease in laser dosage while irradiating cells deep in collagen (∼150 *µ*m), laser irradiation was performed with 3× of the threshold used under monolayer conditions. Thus, a lethal dosage of 0.64 J cm^−2^ (Objective: 40×, NA:0.8, Power: 150 mW, exposure time:0.5 s) was used for all disruption experiments in this work. Here we tested two network designs. First design involves single-layer MLO-Y4 Cellnets in square grid orientation located ∼70 *µ*m inside collagen in the central chamber (Ch#2) of the microfluidic chip (figure 7(A)). On Day 5, focused femtosecond laser was used to lethally injure individual osteocytes (figure 7(B)). Then, Fluo-4 Ca indicator treatment was applied and timelapse fluorescence imaging was used in the presence of fluid flow stimuli to monitor real-time calcium signaling within the disrupted Cellnet. Real-time fluorescence images show an absence of Ca signal and propagation for injured cells within the network. Based on the simplified nomenclature described earlier (figure 7(C) and SI-figure 10), cells 1.2, 1.3 and 2.3 were lethally injured forcing the signals to propagate from cell 1.4 (proximal to stimuli) to cell 2.4 (row 2, column 4) to other cells within the network. Plots shows the time-delays of signaling peaks as the signal travels from cell 1.4 (50 s) to cell 2.4 (56 s) to cell 3.4 (72 s) to cell 2.2 (78 s) (figure 7(D)). In an undisrupted Cellnet, signal would have chosen the shortest path to propagate to cell 2.2 (i.e. from cell 1.2 to cell 2.2), however due to user-defined disruptions, the signal is forced to circumnavigate using the only possible connected path available, as indicated by shifts in the signal intensity peaks and associated time-delays.

Design 2 involves a 2-layer Cellnet in a square grid orientation indicated by ‘*X*’ in figure 8, where the top and bottom layers are located at a depth of 130 *µ*m and 70 *µ*m inside collagen respectively. For pattern ‘*X*’, only cell network located at *z* = 130 *µ*m has direct physical connection to chamber #1 while the cell network located at *z* = 70 *µ*m can only indirectly sense the stimuli through out-of-plane cell-to-cell connections. Fluorescence images show osteocyte morphology (figure 8(A-iii)) and a snapshot of calcium signaling (figure 8(A-iv)) from the imaging plane located at *z* = 70 *µ*m. These results clearly show that there is no direct contact between the flow stimuli applied in Ch#1 and cell network located at *z* = 70 *µ*m.

To compare signal propagation speeds between directly and indirectly connected cell networks, we prepared two network 3D patterns ‘*X*’ and ‘*Y*’ (figures 8(B-i) and (B-ii)). As a control to compare signal propagation speeds, parallel-line pattern *Y* at z = 70 *µ*m directly sense flow-stimuli evoked signaling response while for pattern ‘*X*’, cells located at z = 70 *µ*m can indirectly receive signals from stimuli-evoked cells located at z = 130 *µ*m (figures 8(B-iii) and (B-iv)). Fluorescence image showing a snapshot of calcium signaling within the cell network located at z = 70 *µ*m (figure 8(B-v) and SI-figure 11). (Arrows indicate the signaling direction for both patterns). Cells ‘*a*’ and ‘*b*’ from pattern *Y* and cells ‘*c*’ and ‘*d*’ from pattern *X* are chosen as representative cells, and their signaling responses are compared when subjected to a flow stimulus (figure 8(B-vi)). Peak delays observed in ‘*c*’ and ‘*d*’ as compared to ‘*a*’ and *‘b*’ indicate that pattern X enforces Ca signal propagation from the top-layer (z = 130 *µ*m that directly senses the stimuli applied in Ch#1) to cells in the bottom layer (z = 70 *µ*m). These results show the capability of studying real-time signaling within user defined disrupted cellular networks. The arrows in figure 8(B-vi) indicate the onset of calcium fluorescence in each cell. We chose to mark the onset rather than the peaks because of variations in cell sizes and their temporal signal properties such as peak occurrence and durations. The presence of multiple peaks and non-intuitive signaling occurrences can be attributed to possible heterogeneity in the activation states of individual cells within Cellnets.

## 3. Discussion

All tissues are composed of single cells, yet how tissues sense, respond, and adapt to stimuli cannot be predicted by studying individual cells as higher-level emergent property of the tissue likely rely on complex dynamic interactions between cells. To do this, the field needs new ways to generate tissue-specific 3D single cell networks, apply defined stimuli, and analyze networks’ adaptations. At present, such a technology does not exist. As a result, the link between the individual cells and cell-networks’ (tissue) function remains unclear and difficult to predict. Here, we report a novel technology, coined as Cellnet, to generate normal and disrupted 3D single-cell networks within type I collagen matrix in user-defined architectures and study the real-time signaling of single cells and signal propagation across the entire network. We show that Cellnets, made from many cell types, is highly reproducible and provides control over cell-to-cell connectivity. Use of DLP allows rapid and inexpensive iteration of master molds enabling rapid production of custom chips. We show that real-time calcium signaling of individual cells and signal propagation within Cellnets can be monitored when subjected to biophysical (fluid-flow) and biochemical (ionomycin) stimuli. Moreover, target cells can be fatally injured in a noninvasively, contactless, and sterile manner during active cell culture at defined locations and time-points, and changes in signaling dynamics in custom disrupted networks can be studied. Below we explain some key observations, possible explanations and limitations of our work.

Results in figure 2(C) show that a constant laser dosage causes different lumen widths at different depths within the collagen matrix. However, we do not see any detectable changes in lumen width during vertical or z-scanning (figure 2(D)). This discrepancy can be possibly due to the phenomenon of spherical aberration commonly associated with fs-laser scanning within 3D bulk materials including collagen. The non-linearity of laser-material interactions stretches the focal volume and associated energy deposited along the laser propagation direction (*Z*-direction). Due to a high degree of energy overlap between adjacent regions in the vertical direction combined with small scanning distances in the *z*-direction (<150 *µ*m), we were not able to detect any changes in lumen size during vertical scanning.

In this work, we relied on snapshots of brightfield images to characterize cell migration within microchannel networks. Thus, how cells migrate into and out of lumen templates in collagen or whether cells proliferate within lumen networks was not investigated. We expect both cell migration and proliferation to play key role in both the formation and continued maintenance of single cell network. Time-lapse live imaging studies are needed to obtain insights into single cell dynamics within confined microchannel networks. Also, we observe that cells preferentially position themselves at the nodes, located at the intersection of two lumen, possible due to larger void area generated by intersecting scanning paths during laser-assisted cavitation.

Transport properties of collagen barrier (width = 1 mm) in microfluidic chips were assessed using fluorescent dyes of varying molecular weights (SI-figure 13). Based on the duration of culture in this work (∼7 d), and rapid transport properties of bulk collagen, we expect to see high cell viability for other network architectures, but more experiments are needed to study how 3D geometry influences the viability, migration speeds, and proliferation of cells within the microchannel network. For FITC-Dextran (4 kDa), the transport properties of MLO-Y4-Cellnets and bulk collagen were found to be almost identical. Moreover, no movement of microbeads was observed in cell-laden microchannels implying that advection was absent in chamber #2. This makes sense as cell-occupied microchannels provide minimal gaps for dye or microbeads to flow. Future work will seek to answer how 3D geometry influences the viability, migration speeds, and proliferation of cells within the microchannel network.

In this work, we define biophysical flow stimulation as the effect generated by manual pipetting 150 *µ*l media into the inlet of chamber #1 of the chip. Results show that collagen barrier between adjacent posts is deformed by ∼5 *μ*m and this lasts for a duration of about 2.75 s (SI-figure 14 and videos 3–5). Moreover, video 6 shows that addition of 5 *μ*l of ionomycin in chamber #1 does not cause any deformation of collagen barrier, which implies that recorded calcium signals within Cellnet is solely due to biochemical and not mechanical stimulation. Further experiments with high-speed camera and elastic modeling of viscoelastic collagen needs to be performed to precisely characterize the shear stresses experienced by the cells in different network architectures.

Here, we choose rat tail collagen type I as our model ECM matrix, due to its widespread use in the field. Our protocol can repeatably generate microchannel network of user-defined diameters which upon cell-seeding reliably self-organize into viable single-cell networks in collagen matrix. We also show that Cellnet can be generated from other commonly used ECM analogs such as bovine collagen, synthetic PEGDA 6k, and GelMA (SI-figure 12). We found that for identical laser dosage, hydrogels with higher crosslinking density and stiffness (reduced water content) produced more uniform and superior resolution microchannels. However, complete understanding of fs-laser induced cavitation within hydrogels remains challenging as multiple laser properties (intensity, duration, scan speed, pulse width, frequency, repetition rate, focal volume, and optical setups) and hydrogel properties (two-photon absorption, composition, degree of hydration) are known to influence fs-laser and matrix interactions. It is known that fs-laser irradiation within hydrogels induces multiphoton ionization which results in direct ablation of the matrix confined within the ellipsoidal focal volume, and above a certain laser dosage, leads to the formation of spherical cavitation microbubbles. A schematic representation of this process is shown in SI figure 3 and video 7. However, based on hydrogels’ physical, biochemical, structural, and optical properties, two-photon absorption cross-section could vary significantly. Elucidating the complex interplay between laser setups, matrix properties, and cavitation dynamics lies beyond the scope of this work and will require further investigation.

Cellnet generated within multi-chambered microfluidic chip is ideally suited to apply defined mechanical forces to mimic various biophysical cues experience by cell-networks (tissues) *in vivo*. On-demand printing of Master molds allows rapid iterations in channel geometry or chips designs to include inlets and outlets of various sizes and enable its integration with syringe pumps or peristaltic pumps to apply custom mechanical stimuli in form of flow or pressure variations. Compressive forces can be also applied from the top of PDMS chip during active cell cultures.

Scalability of Cellnet for large scale studies face several challenges. Cellnet requires use of expensive femtosecond lasers and related knowledge about optical engineering. The total duration to make one Cellnet includes 2–3 d for developing a PDMS chip with crosslinked collagen followed by 5–10 min of laser scanning time to generate all 3D microchannel networks architectures reported in this work. Since Cellnet are generated within microfluidic chips, the overall depth of light penetration was not a constraint in this work. However, scaling up in vertical direction or generating large-volume network will not be feasible with this approach. For scaling up, the rate limiting step will be the serial laser scanning process which is a function of both network design and matrix properties. This can be improved by use of high energy lasers, automated alignment and stage movements, galvanomirrors, and microlens arrays. The time to make master molds can also be decreased by printing an array of positive molds in the same print. Further, this work is limited to ECMs that are semi-transparent to the wavelength of the fs laser to ensure no attenuation or scattering during laser-assisted cavitation process. Also, for each cell type, target lumen size must be optimized to ensure single-cell occupancy within microchannel networks. This work exclusively focuses on demonstrating a new technology, but more work is needed to study the impact of laser-assisted cavitation on the viscoelastic properties of hydrogels.

To test the capability of studying real-time signaling within Cellnets, we choose osteocytes as our model cells, due to our groups’ prior experience with bone tissue engineering [30]. Like many tissues, in bone, stimuli evoke calcium signals within 3D, organized, single-cell osteocyte networks while signal disruptions have been linked to many pathologies. Similar to other cell types, single-cell osteocyte networks have also been generated using micropatterned cell-adhesive SAMs or micro-chambers [31, 32], or two-photon laser based modifications [9, 28], however generating single-cell network in 3D remain difficult [33–36]. As a result, culture cells within collagen or fibrin matrices remain the ‘gold standard’ to generate 3D cell networks, however the randomly organized cell-cell connections with poor reproducibility makes a systematic study about single cell and its relationship to network signaling difficult [37–48]. In contrast, structurally defined 3D MLO-Y4 (osteocyte) Cellnets simplify image processing and enable accurate mapping of how single cells respond to various types of stimuli and their contributions to changes in signaling wavefronts within the networks. In this work, we used manual analysis to track signaling changes within Cellnets, but considering the large amount of signaling data generated from a variety of stimuli conditions, new automated mapping tools will be needed to study simultaneous activity behavior in different parts of Cellnets and elucidate insights into signaling mechanisms.

Previously, our group and others have used femtosecond lasers to modify local properties within a 3D cell-laden hydrogel matrix to guide the alignment of single cells. For instance, our group showed laser based densification of partially crosslinked GelMA was used as guidance cues to align encapsulated cells in 3D [15]. Another study used modified PEGDA hydrogels to generate cell network [28]. In both these cases, native ECM like collagen cannot be used due to the requirement of the photo-sensitive hydrogels to enable laser-based biochemical or biophysical modifications. Both laser processing conditions and hydrogel properties must be optimized to maximize viability of encapsulated cells; this enforces strict constraints on hydrogel type and processing conditions and limits its utility in the field. Laser scanning also generates reactive oxygen species (ROS) which decrease cell viability by interfering with its metabolism. Since ROS generation is strongly dependent on applied laser dosage and the material photosensitivity, new semi-synthetic hydrogels with better photosensitivity are needed to satisfy the contrasting requirements of rapid hydrogel modifications (either ablation [7, 9] or degradation [6, 8]) while maintaining high cell viability. With Cellnet, a user-defined templated in collagen is generated before cells are seeded/pipetted in target microfluidic chambers. This decoupling provides flexibility to generate 3D cell networks in any bioink including native and unmodified ECM like collagen which in turn results in close to 100% cell viability as laser scanning is not performed in the presence of cells. Although we focus on type I collagen (4 mg ml^−1^) as our model ECM, we envision Cellnet to be an ECM- and cell-agnostic technology that can be broadly used to design tissue-specific Cellnets for a range of applications.

Lastly, Cellnet could also emerge as an ideal tool to study signaling heterogeneity, a potentially important yet unstudied phenomenon. Previously, we and others have shown that self-assembled and randomly connected osteocyte networks exhibit spatial variation and temporal heterogeneity of calcium waveforms when subjected to fluid flow stimuli [30]. Due to difficulty in identifying individual cells in self-assembled and randomly organized networks, the relationship between cell connectivity, stimulus conditions, and signal heterogeneity cannot be studied. In this work we use calcium signal as a proxy to monitor real-time signaling of individual osteocytes within spatially organized 3D single cell networks. Cellnets within microfluidic chips provide systematic application of biophysical, biochemical or injury stimuli that could be used to assess single cell signaling heterogeneity within other cell types. Overall, the modular nature of Cellnet allows ‘swapping in’ relevant cell types, ECM composition, network designs, optical tracers for other signals (NO, ROS, ATP) and/or fluorescent reporters or inhibitor drugs and acquire new knowledge using standard imaging and culture methods. In the future, transfected variants of cell lines with genetically encoded calcium indicators can facilitate fluorescence imaging without exogenous labels and allow us to study the dynamic evolution of Cellnets over time. The simple and easy to use cell seeding strategy to generate Cellnets will enable broad utility and adoption in the field to test new hypothesis across cell, tissue, or stimulus types and develop personalized tissue-specific s the study of tissue-scale system biology by linking individual cells and tissue networks’ function thereby elucidating higher-level emergent property.

## Data Availability

All data that support the findings of this study are included within the article (and any supplementary files).

## Materials and methods

Please refer to the SI section for details.

## Impact statement

New technology to generate 3D single-cell interconnected and disrupted networks within natural extracellular matrix in custom architectures.
